# Characterization of Highbush Blueberry (*Vaccinium corymbosum* L.) Anthocyanin Biosynthesis Related MYBs and Functional Analysis of *VcMYB* Gene

**DOI:** 10.3390/cimb45010027

**Published:** 2023-01-03

**Authors:** Yongyan Zhang, Dingquan Huang, Bin Wang, Xuelian Yang, Huan Wu, Pengyan Qu, Li Yan, Tao Li, Chunzhen Cheng, Dongliang Qiu

**Affiliations:** 1 College of Horticulture, Fujian Agriculture and Forestry University, Fuzhou 350002, China; 2 College of Horticulture, Shanxi Agricultural University, Jinzhong 030801, China; 3 College of Agriculture, Fujian Agriculture and Forestry University, Fuzhou 350002, China; 4 College of Agriculture, Guizhou University, Guiyang 550025, China; 5 Fruit Research Institute, Fujian Academy of Agricultural Sciences, Fuzhou 350013, China

**Keywords:** MYB, blueberry, anthocyanin, gene expression, expression regulation

## Abstract

As one of the most important transcription factors regulating plant anthocyanin biosynthesis, MYB has attracted great attentions. In this study, we identified fifteen candidate anthocyanin biosynthesis related MYB (ABRM) proteins, including twelve R2R3-MYBs and three 1R-MYBs, from highbush blueberry. The subcellular localization prediction results showed that, with the exception of VcRVE8 (localized in chloroplast and nucleus), all of the blueberry ABRMs were nucleus-localized. The gene structure analysis revealed that the exon numbers of the blueberry *ABRM* genes varied greatly, ranging between one and eight. There are many light-responsive, phytohormone-responsive, abiotic stress-responsive and plant growth and development related *cis*-acting elements in the promoters of the blueberry *ABRM* genes. It is noteworthy that almost all of their promoters contain light-, ABA- and MeJA-responsive elements, which is consistent with the well-established results that anthocyanin accumulation and the expression of *MYBs* are influenced significantly by many factors, such as light, ABA and JA. The gene expression analysis revealed that *VcMYB*, *VcMYB6*, *VcMYB23*, *VcMYBL2* and *VcPH4* are expressed abundantly in blueberry fruits, and *VcMYB* is expressed the highest in the red, purple and blue fruits among all blueberry *ABRMs*. *VcMYB* shared high similarity with functionally proven *ABRMs* from many other plant species. The gene cloning results showed that *VcMYB* had three variable transcripts, but only the transient overexpression of *VcMYB-1* promoted anthocyanin accumulation in the green fruits. Our study can provide a basis for future research on the anthocyanin biosynthesis related *MYBs* in blueberry.

## 1. Introduction

Anthocyanins, which are natural polyphenols that are widely found in many foods, possess many biological and health-beneficial abilities, such as anti-inflammatory, visual-improving and so on [1,2,3,4]. All of these abilities are achieved through their high antioxidant activity [5,6] and some other biological effects, such as their anti-proliferative effect [7], hepatoprotective effect [8] and anti-depressant behavior [9].

Blueberries are one of the richest sources of anthocyanins among common fruits [10], and the bio-availabilities of blueberry anthocyanins have been extensively investigated [11,12]. During the last two decades, a large number of studies have been conducted on anthocyanin metabolism [13,14,15,16,17,18,19,20,21,22], and some anthocyanin biosynthesis related structural genes (such as *cinnamic acid 4-hydroxylase* (*C4H*) [13], *flavanone-3-hydroxylase* (*F3H*) [14], *dihydroflavonol reductase* (*DFR*) [15], *anthocyanidin synthase* (*ANS*) [16]) and regulatory transcriptional factors (such as genes encoding MYBs and bHLHs [17,18,19,20,21,22]) were identified or functionally verified in blueberry. 

The MYB transcription factor plays an important role in regulating the spatio-temporal expression of anthocyanin biosynthesis related structural genes and anthocyanin accumulation, and is one of the most important and widely investigated transcription factors involved in anthocyanin biosynthesis regulation [17,22]. The first plant MYB gene, *C1*, was discovered from maize [23], whose encoded protein was required for anthocyanin biosynthesis in the aleurone layer [24]. Since then, *MYBs* have been isolated from many plant species. Most MYB proteins contain conserved MYB domain/domains at their N-terminus. According to the number of their MYB domains, MYBs can be further classified into four types, i.e., 1R-MYB/MYB-related, R2R3-MYB, 3R-MYB and 4R-MYB. It is noteworthy that most of the reported anthocyanin biosynthesis related MYBs (ABRMs) were R2R3-MYBs, followed by 1R-MYB/MYB-related MYBs [25]. In blueberry, most of the reported anthocyanin metabolism regulatory transcription factors were MYBs [26]. For example, the up-regulated expression of *VcMYB21* and *VcR2R3MYB* was confirmed to be associated with the UV-B-induced anthocyanin accumulation in blueberry pericarp [27]; VcMYB21 played a negative regulatory role in anthocyanin accumulation [28]; the transient overexpression of blueberry *MYBA* in an *Antirrhinum majus MYB* mutant restored the anthocyanin accumulation, and its co-expression with a heterologous *bHLH* could induce anthocyanin accumulation in tobacco leaves [29]; the pink fruit mutation phenotype of ‘Pink Lemonade’ was caused by the mutation of *MYB1* [20]; the expression of *VcUFGT* in highbush blueberry was positively regulated by VcMYBA1 and negatively regulated by VcMYBC2 [30]. 

Although some studies have been conducted on blueberry *ABRMs* [31,32], their applications are restricted as almost all these reported *ABRMs* were obtained based on *de novo* assembled transcriptome data. The publication of the draft genome of blueberry [33,34] greatly facilitated the genome-wide identification of the functional and regulatory genes involved in anthocyanin biosynthesis [35]. Based on the genome data, Wang et al. [31] identified a total of 229 *MYB* members that could be further divided into 23 subfamilies, but they did not focus on the anthocyanin biosynthesis related members; Zhao et al. [17] identified 11 MYBs from blueberry fruits through homologous protein searching, using Arabidopsis, apple, grape and strawberry MYBs belonging to the MBW complex as queries. Recently, many *ABRMs* have been identified and functionally approved in various plant species, such as Arabidopsis [36], *Helianthus tuberosus* [37], monkeyflower [38], *Eutrema salsugineum* [39], *Freesia hybrida* [40], grape hyacinth [41], apple [42] and so on. In this study, for the exploration and characterization of blueberry ABRMs, we identified the blueberry ABRMs by homologous searches against the blueberry protein data provided by the highbush blueberry genome project using the reported ABRMs from some other plant species as queries, characterized their sequences, and investigated their corresponding genes’ expression patterns in blueberry fruits at five different ripening stages, based on our previously obtained transcriptome data and quantitative real time PCR (qRT-PCR) analysis. Moreover, the function of *VcMYB* (VaccDscaff1486-snap-gene-0.3), whose encoded protein showed high similarity with ten functionally proved ABRMs from other plant species (including Arabidopsis AtMYB114, AtMYB90, AtMYB75 and AtMYB113, *H. tuberosus* HtMYB2, monkeyflower PELAN, *E. salsugineum* EsMYB90, *F. hybrida* FhPAP1, grape hyacinth MaAN2 and apple MdMYB10), was further studied by transient overexpression in young blueberry fruits. The results obtained in this study will provide a foundation for the functional analysis and applications of blueberry anthocyanin biosynthesis related *MYB* genes, and will lay the foundations for research on the high-anthocyanin aimed blueberry breeding in the future.

## 2. Materials and Methods

### 2.1. Plant Materials

The plant materials used in this study were the fruits of four-year-old southern highbush blueberry ‘FL03’ at five different stages (green fruit (GF), pink fruit (PiF), red fruit (RF), purple fruit (PF) and blue fruit (BF)) [22]. After harvesting, the fruits were quickly taken back to the laboratory, washed with distilled water three times, immediately frozen in liquid nitrogen after draining the fruit surface with sterilized filter paper, and stored in the refrigerator at −80 °C for further use.

### 2.2. Identification of ABRM Proteins in Blueberry

The gDNA, cDNA and protein sequence files of the blueberries were downloaded from https://www.vaccinium.org/analysis/49 (accessed on 3 March 2021). To identify ABRM proteins in the blueberries, homologous protein sequence alignment was performed against the blueberry protein data, using recently reported ABRM protein sequences as queries with e-value ≤ 1 × 10^−5^ and similarity >50% as criteria. The screened sequences with the highest bit score were selected as candidate MYBs and were named according to their homologous proteins sharing the highest similarity with them. One exception is that one candidate MYB (VaccDscaff1486-snap-gene-0.3) was named as VcMYB to distinguish it from blueberry VcMYBA (MH105054) [32]. A phylogenetic tree was constructed by Maximum Likelihood method using MEGAX (Possion mode, complete deletion, and 1000 bootstrap values) to show the relationships among the blueberry ABRMs and the reported ABRMs from some other plants.

### 2.3. Bioinformatic Analysis of Blueberry ABRM Genes and Their Encoded Proteins

The physicochemical properties, conserved motifs (motif number set as 10) and domains, as well as the subcellular localization of the blueberry MYB proteins, were analyzed according to the method described by Zhang et al. [22]. Gene structure analysis was performed using GSDS (http://gsds.gao-lab.org/, accessed on 3 March 2021). The ClustalW program in MEGA 6.06 software was used for multiple protein sequence alignment, and the alignment results were incorporated into MEGA 6.06 for the construction of the phylogenetic tree using the neighbor-joining method under the criteria of the Poisson model, complete deletion and bootstrap value = 1000. The 2000 bp sequences to upstream the start codon of the *MYBs* were extracted from the blueberry genome database and considered as promoter sequences, and were then subjected to *cis*-acting element analysis using PlantCARE (http://bioinformatics.psb.ugent.be/webtools/plantcare/html/, accessed on 3 March 2021).

### 2.4. Gene Expression Analysis

In our previous study, we sequenced the transcriptome of ‘FL03’ blueberry fruits at five different ripening stages (GF, PiF, RF, PF and BF). To study the expression patterns of these identified blueberry *ABRMs*, their FPKM (Fragments Per Kilobase of exon model per Million mapped fragments) values were first extracted from the transcriptome data and transformed into Log_2_(FPKM + 1) for heatmap drawing using TBtools software [43]. 

To validate the expression of the blueberry *ABRMs*, four highly expressed *ABRMs*, including *VcMYB6*, *VcMYB23*, *VcMYBL2* and *VcMYB*, were selected and subjected to quantitative real time PCR analysis. Primers were designed according to their CDS sequences using Primer 3.0 (Table 1). A Trizol RNA Extraction Kit (TaKaRa, Dalian, China) was used to isolate the total RNA from the blueberry fruits at five different ripening stages. Then, high quality RNA was used for cDNA synthesis using TransScript All-in-One First-Strand cDNA Synthesis SuperMix for qPCR (One-Step gDNA Removal) kit (TransGen, Beijing China). The qRT-PCR reactions were performed on a Bio-Rad CFX96^TM^ real-time quantitative fluorescent PCR instrument using a TB Green Premix Ex Taq II (Tli RNaseH Plus; TaKaRa, Dalian, China) kit according to the manual. The relative expression levels of the selected blueberry *ABRM* genes in the fruits at different ripening stages were calculated using the 2^−ΔΔCt^ method with *GAPDH* (Genbank ID: AY123769) as the internal reference gene [22]. Three biological and three technical replications were made for the qRT-PCR analysis of the selected genes.

### 2.5. Gene Cloning, Vector Construction and Transient Overexpression Analysis

A RevertAid First-strand cDNA synthesis Kit (Thermo Scientific, Shanghai, China) was used to synthesize the cDNA for gene cloning. The primers for *VcMYB* gene amplification were designed using Primer 3 (Table 1). The 25 μL PCR system contained 1 μL cDNA, 1 μL each of the forward and reverse primer, 12.5 μL 2 × Green mix and 9.5 μL ddH_2_O. The PCR conditions were set as follows: predenaturation at 95 °C for 3 min; 35 cycles of denaturation at 95 °C for 30 s, annealing at 59 °C for 30 s, and extension at 72 °C for 2.5 min; and final extension at 72 °C for 8 min. The electrophoresis detection results showed that there were three variable transcripts for *VcMYB*. The PCR products of the three transcripts were separately gel extracted, ligated into an 18-T vector and transformed into *Escherichia coli* DH5a. Positive clones were selected and sent to Beijing Liuhe Huada Gene Technology Co., Ltd. (Beijing, China) for sequencing verification. According to the sequencing results, the primers for the *VcMYB* gene vector construction were further designed and used for the amplification of the three variable transcripts (Table 1). The cloned fragments were ligated into the pBI123 vector using the Ready-to-use Seamless Cloning Kit (Sangon Biotech, Shanghai, China), and transformed into *Agrobacterium tumefaciens* GV3101. *A*. *tumefaciens* GV3101 carrying empty pBI123 (as control), pBI123-*VcMYB-1*, pBI123-*VcMYB-2* and pBI123-*VcMYB-3* vectors were cultured in the dark at 180 rpm and 28 °C until OD_600_ = 0.6~0.8 they were centrifuged at 5000 rpm for 4 min, resuspended using an MES solution (2-(N-Morpholino) and ethanesulfonic acid hydrate) solution (containing 10 mM MES, 10 mM MgCl_2_·6H_2_O and 200 μM acetosyringone) to a final concentration of OD_600_ = 0.6. Then, they were injected into the green fruits (at about 15 days post flowering (dpf), to further confirm the function of *VcMYB-1*, green fruits at 20 dpf were also used) of the ‘Legacy’ blueberry, cultured in the dark for 1 day and removed to normal light for fruit color change observation. At three days post treatment, the colour parameters (L*, a* and b* values) of the fruit pericarps were measured using a CR8 Portable Colorimeter (Shenzhen Threenh Technology Co., Ltd., Shenzhen, China), and the anthocyanin was extracted using the acidified ethanol [44] from the blueberry fruit pericarps. The anthocyanin content in the blueberry pericarps was measured and calculated according to the method of Zhuang et al. [45].

## 3. Results

### 3.1. The Identified Blueberry ABRMs

Through homologous protein searching, fifteen candidate blueberry ABRM proteins were identified (Table 2). The phylogenetic analysis results revealed that all of these blueberry ABRM proteins shared a close relationship with the query homologous proteins from other plants (Supplementary Figure S1). Among them, VcMYB shared high similarity, up to 93.40%, with the reported blueberry anthocyanin biosynthesis regulatory VcMYBA [32]. Moreover, it shared more than 50% sequence similarities with ten known ABRMs, including Arabidopsis AtMYB114 (74.36%), *H. tuberosus* HtMYB2 (62.25%), monkeyflower PELAN (59.87%), *E. salsugineum* EsMYB90 (59.35%), Arabidopsis AtMYB90 (PAP2) (58.97%) and AtMYB75(PAP1) (58.71%), *F. hybrida* FhPAP1 (56.86%), grape hyacinth MaAN2 (56.86%), Arabidopsis AtMYB113 (56.77%) and apple MdMYB10 (54.36%). VcMYB12 shared the highest similarity with Arabidopsis AtMYB12 (66.86%), followed by AtMYB12 and AtMYB111. The similarities of VcMYB123 with kiwifruit AcMYB123 and apple MYB9 were both higher than 70% (72.33% and 71.43%, respectively). VcMYBL2 shared the highest similarity with apple MdMYBL2 (65.29%), followed by eggplant SmelMYBL1 (51.61%). VcMYB6 shared the highest similarity with apple MdMYB6 (55.26%). VcPL shared the highest similarity with rice OsPL (75.19%). VcMYB24L shared the highest similarity with apple MdMYB24L (75.00%). VcRVE8 shared the highest similarity to *Pyrus bretschneideri* PbRVE8 (68.22%). VcCPC shared the highest similarity with Arabidopsis AtCPC (57.41%). VcMYBC1, VcMYB23, VcMYB340, VcPH4, VcMYBATV and VcMYB85 shared the highest similarity with kiwifruit AaMYBC1, apple MdMYB23, sweet potato IbMYB340, citrus CsPH4, tomato SlMYBATV and millet SiMYB85, respectively. 

### 3.2. Physiobiochemical Properties and Sequence Characteristics of Blueberrry ABRMs

**Figure 1:**
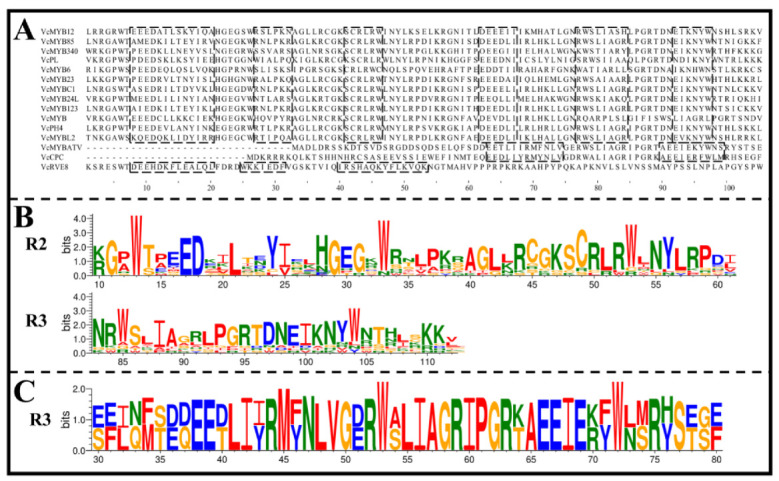
Repeat (R) domains sequences in blueberry ABRM proteins. (**A**): The R domain sequences of blueberry ABRM proteins. Sequences in dashed boxes represent the helix sequences in R domains; (**B**): Sequence logos for the R2 and R3 domain of R2R3-MYB type blueberry ABRMs; (**C**): Sequence logo for the R3 domain of 1R-MYB type blueberry ARBMs.

The identified blueberry ABRM proteins consisted of between 73 and 365 aa, with molecular weights ranging between 8407.08 and 40,095.85 Da, and isoelectric points (pI) ranging between 4.31 and 9.72. Their instability coefficients ranged between 36.10 and 79.67. All of these proteins were predicted to be hydrophilic proteins. The subcellular localization prediction results showed that all of these blueberry ARBMs were nucleus-localized, with the exception of VcRVE8 (localized to both chloroplast and nucleus) (Table 3). By analyzing the amino acid sequences, we found that VcMYB12, VcMYB85, VcMYB340, VcPL, VcMYB6, VcMYB23, VcMYBC1, VcMYB24L, VcMYB123, VcMYB, VcPH4 and VcMYBL2 have conserved R2 and R3 domains, indicating that they belong to the R2R3-MYB (2R-MYB). The remaining three ARBMs belong to 1R-MYB, of which VcMYBATV and VcCPC contain a R3 domain, and VcRVE8 contains a R1/2 domain (Figure 1).

### 3.3. Conserved Motifs in Blueberry ABRM Proteins and Gene Structures of Their Corresponding Genes

**Figure 2:**
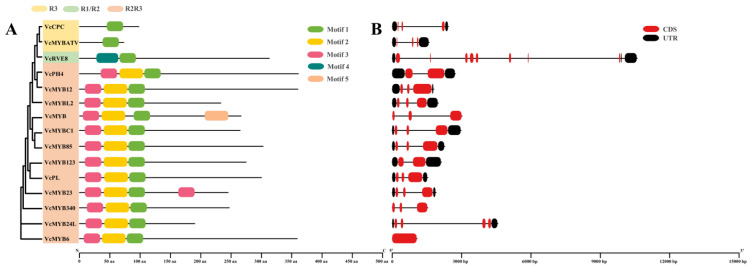
Conserved motifs distributions in blueberry ABRM proteins (**A**) and gene structures of their corresponding genes (**B**).

In total, we identified five conserved motifs from the blueberry ABRM proteins (Figure 2A). Among these motifs, Motif1 was found in all of the ABRMs. The R2R3 type blueberry ABRMs all contained Motif1~3, VcMYB23 contained two Motif3, and VcMYB contained an extra Motif5. VcPCP and VcMYBATV contained only one Motif1, VcRVE8 contained only one Motif1 and one Motif4.

The gene structure analysis results showed that the exon numbers of the blueberry *ARBM* genes ranged between one and eight (Figure 2B). *VcRVE8* had the largest number of exons with eight, *VcMYB24L* had four exons, *VcPH4* and *VcMYB123* contained two exons, and *VcMYB6* had only one exon. All the other blueberry *ABRMs* contained three exons.

### 3.4. Cis-Acting Elements in Promoters of Blueberry ABRM Genes

**Figure 3:**
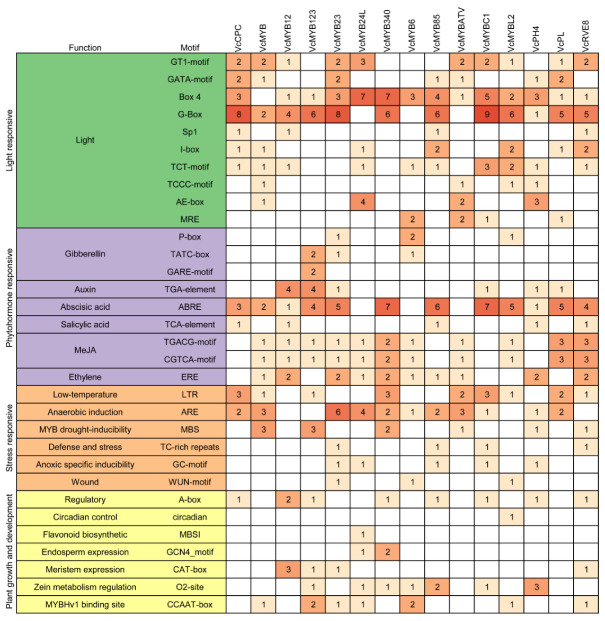
The identified *cis*-acting elements in the promoters of blueberry anthocyanin biosynthesis related *MYB* genes.

We further analyzed the *cis*-acting elements in the promoters of the blueberry *ABRM* genes. The results showed that there were many light-responsive, phytohormone-responsive, stress-responsive, and growth and development related elements in their promoters (Figure 3). In total, we identified ten types of light-responsive elements in their promoters. All of the blueberry *ABRMs*, with the exception of *VcMYB*, contained a light-responsive Box4 element in their promoter regions, and the promoters of 12 blueberry *ABRM* genes (except *VcMYB24L*, *VcMYB6* and *VcMYBATV*) contained the light responsive G-box elements (Figure 3).

Nine types of phytohormone-responsive elements, including three gibberellin (GA)-responsive (P-box, TATC-box and GARE-motif), two methyl jasmonate (MeJA)-responsive (TGACG-motif and CGTCA-motif), one auxin-responsive (TAG-element), one abscisic acid (ABA)-responsive (ABRE), one salicylic acid (SA)-responsive (TCA-Element), and one ethylene-responsive elements (ERE), were found in the promoters of blueberry *ABRM* genes (Figure 3). With the exception of *VcMYB24L*, *VcMYB6* and *VcMYBATV*, all the other *ABRMs*’ promoters contained ABA-responsive ABRE elements. With the exception of *VcCPC*, *VcMYB85*, *VcMYBC1* and *VcPH4*, the promoters of all the other *ABRM* genes contained the MeJA-responsive TGACG-motif and CGTCA-motif elements. With the exception of *VcPCP*, *VcMYB123*, *VcMYBC1*, *VcMYBL2* and *VcPL*, all the other *ABRMs*’ promoters contained ethylene-responsive ERE elements. In addition, auxin-, SA- and GA-responsive elements were found in the promoters of six, five and four *ABRMs*, respectively.

In total, six kinds of stress-related elements, including low temperature-responsive element LTR, anaerobic-induction related element ARE, MYB drought-inducibility related element MBS, defense and stress related element TC-rich elements, anoxic specific inducibility related element GC-motif and wounding related element WUN-motif, were identified in the blueberry *ABRM* promoters (Figure 3). Moreover, there were eleven, nine, six, five, four and three *ABRMs* contained ARE, LTR, MBS, GC-motif, TC-rich repeats and WUN-motif in their promoters, respectively.

Additionally, we also identified many growth and development related *cis*-acting elements in the promoters of the blueberry *ABRM* genes (Figure 3). Eight *ABRMs* contained the regulatory A-box element in their promoters; seven *ABRMs*’ promoters contained the zein metabolism regulation related O2-site element; seven *ABRMs*’ promoters contained the MYBHV1-binding site element CCAAT-box; four *ABRMs*’ promoters contained the meristem expression related element CAT-box; the promoters of *VcMYB24L* and *VcMYB340* contained the endosperm expression related GCN4_motif element; the promoter of *VcMYBL2* contained the circadian control related element. Moreover, the *VcMYB24L* promoter specifically contained the flavonoid biosynthetic related MBSl element.

### 3.5. Protein and Protein Interaction Analysis of Blueberry ABRM Proteins

**Figure 4:**
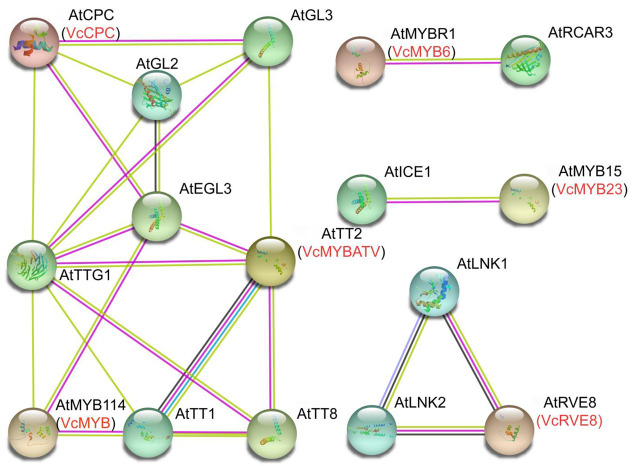
The predicted protein-protein interaction network for blueberry anthocyanin biosynthesis related MYBs based on the Arabidopsis protein database using STRING. At: *Arabidopsis thaliana*; Vc: *Vaccinium corymbosum*.

Based on the Arabidopsis protein database, the STRING software was used to predict the interacting proteins of blueberry ABRMs (Figure 4). The results showed that VcCPC, VcMYB, VcMYB6, VcMYBATV, VcMYB23 and VcRVE8 were homologous protein of AtCPC (At2G46410), AtMYB114 (At1G66380), AtMYBR1 (At5G67300), AtTT2 (At5G35550), AtMYB15 (At3G23250) and AtRVE8 (At3G09600), respectively. AtPCP interacts with AtGL3 (At5G41315), AtEGL3 (At1G63650), AtGL2 (At1G79840) and AtTTG1 (At5G24520). AtMYB114 interacts with AtEGL3 (At1G63650), AtTT1 (At1G34790) and AtTTG1 (At5G24520). AtTT2 interacts with AtTT1 (At1G34790), AtTT8 (At4G09820), AtEGL3 (At1G63650), AtTTG1 (At5G24520) and AtGL3 (At5G41315). AtMYBR1 (At5G67300) interacts with AtRCAR3 (At5G53160). AtMYB15 (At3G23250) interacts with AtICE1 (At3G26744). And AtRVE8 interacts with AtLNK1 (At5G64170) and AtLNK2 (At3G54500).

### 3.6. Expression Analysis of Blueberry ABRM Genes

**Figure 5:**
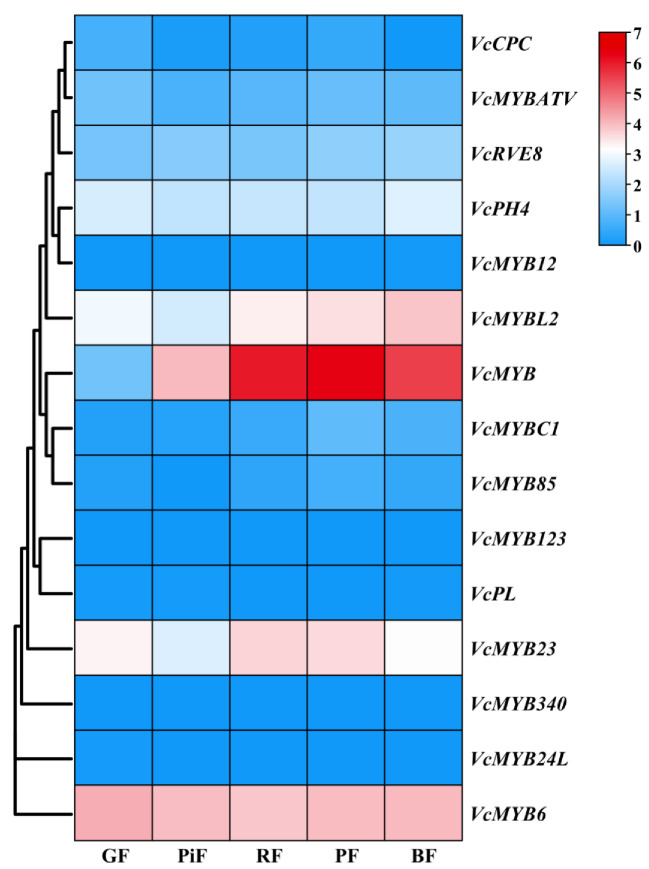
Expression analysis of blueberry *ABRM* genes in fruits at five different ripening stages based on transcriptome data. GF: green fruit; PiF: pink fruit; RF: red fruit; PF: purple fruit; BF: blue fruit. Log_2_(FPKM + 1) values were used for heatmap drawing.

According to our transcriptome data, the expression patterns of the blueberry *ABRM* genes in fruits at different ripening stages were studied. It was found that *VcMYB6*, *VcMYB23*, *VcMYB*, *VcMYBL2* and *VcPH4* are expressed highly in blueberry fruits, but other *ABRMs*, such as *VcMYB12*, *VcMYB123*, *VcPL*, *VcMYB24L* and *VcMYB340*, showed either low expression (FPKM < 4) or are not expressed in the fruits (Figure 5). The expression level of *VcMYB* was low in GF (FPKM < 2), gradually increased in PiF (FPKM > 10), and then maintained at an abundant level in RF, PF and BF (FPKM > 40). The expression levels of *VcMYB* in RF and PF were both higher than that in BF. Moreover, its expression in the late three stages was found to be the highest among all the blueberry *ABRMs* and very significantly higher than that in GF and PiF, indicating that it might play an important role in regulating anthocyanin biosynthesis, particularly at the late fruit ripening stages. The expression level of *VcMYB6* in fruits at all stages was high (FPKM > 10), and its expression level changed slightly during blueberry fruit ripening. The expression of *VcMYB23* was the highest in RF and PF. *VcMYBL2* showed a ‘fall-rise’ expression pattern, and its expression level in PiF was the lowest, but there was no significant expression difference at the late three ripening stages. *VcPH4* also showed a ‘fall-rise’ expression profile during fruit ripening, but no significant difference was found among the fruits at different ripening stages.

**Figure 6:**
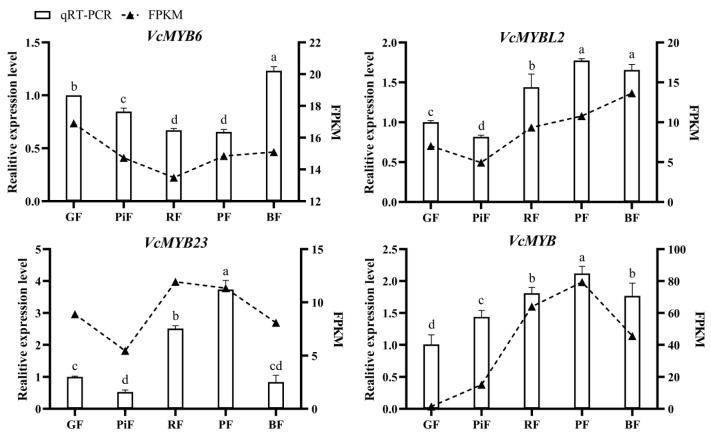
Quantitative real time PCR results of four selected blueberry *ABRM* genes. GF: green fruit; PiF: pink fruit; RF: red fruit; PF: purple fruit; BF: blue fruit. Different letters above columns represent significant difference at *p* < 0.05 level.

For the validation of the expression changes of the blueberry *ABRM* genes in fruits during ripening, quantitative real time PCR (qRT-PCR) analysis of four selected genes, including *VcMYB6*, *VcMYB23*, *VcMYBL2* and *VcMYB*, was performed. The results showed that the expression change patterns of these blueberry *ABRMs* during fruit ripening were mostly consistent with our transcriptome data (Figure 6), indicating that our transcriptome data is believable. Among them, the expression of *VcMYB* was found to be the lowest in GF, and its expression in PiF, RF, PF and BF was approximately 1.44-fold, 1.79-fold, 2.1-fold and 1.75-fold of GF, respectively.

### 3.7. Transient Overexpression Analysis of Three VcMYB Variable Transcripts

**Figure 7:**
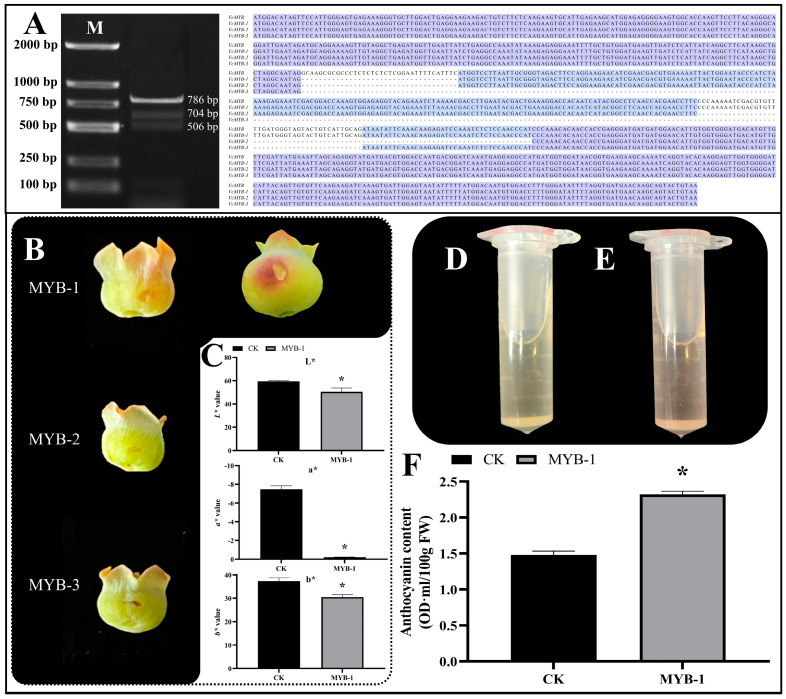
*VcMYB* gene has three variable transcripts and only the transient overexpression of *VcMYB-1* promotes the anthocyanin accumulation in blueberry fruits. (**A**): The three amplified transcripts of *VcMYB*, and sequence alignment results of their nucleotide sequences together with their reference *VcMYB* (VaccDscaff1486-snap-gene-0.3). (**B**): Transient overexpression of *VcMYB* variable transcripts in green blueberry fruits. For fruits of the MYB-1 group, the left one is green fruit at 15 days post flowering, and the right one is green fruit at 20 days post flowering (**C**): Colour parameters (L*, a* and b* values) of fruit pericarps at three days post treatment. (**D**): Anthocyanin extract solution of CK group. (**E**): Anthocyanin extract solution of MYB-1 group. (**F**): Anthocyanin content in blueberry fruit pericarps. The ‘*’ above columns in C and F represents significant difference at *p* < 0.05 level.

Given the high similarity with more than ten reported *ABRM* genes and its high expression in fruits, particularly at the late ripening stages, *VcMYB* was proposed to play a key role in regulating blueberry anthocyanin biosynthesis. To confirm its function, the gene was successfully cloned by reverse transcription PCR (RT-PCR). The electrophoresis detection results showed that this gene had three variable transcripts, which were all shorter than the reference cDNA sequence (VaccDscaff1486-snap-gene-0.3) (Figure 7A). The sequencing results showed that the lengths of these three transcripts were 786 bp, 704 bp and 568 bp, respectively, and they were termed as *VcMYB-1*, *VcMYB-2* and *VcMYB-3*, in descending order of sequence length. 

The three transcripts were separately inserted into the pBI123 vector, transformed into *A. tumefaciens* GV3101, and then transiently overexpressed in the young blueberry fruits. The results showed that only the transient overexpression of *VcMYB-1* triggered anthocyanin accumulation in the young blueberry fruits (Figure 7B). For the green blueberry fruits, at approximately 15 days post-flowering (dpf), at three days post-treatment, the areas injected with *A. tumefaciens* GV3101 carrying *VcMYB-1* became purple-red, and the L*, a* and b* values of the fruit pericarps overexpressing *VcMYB-1* were all significantly lower than the control check group (CK) (Figure 7C). For the green fruits, at about 20 dpf, at three days post-treatment, the injected areas became much redder than the fruits at 15 dpf. By measuring the anthocyanin content, we found that the anthocyanin content in the *VcMYB-1* overexpressing fruit pericarps was approximately 1.56-fold of the CK (Figure 7D–F). However, no obvious pigmentation was found in the areas injected with *A. tumefaciens* GV3101 carrying *VcMYB-2* and *VcMYB-3*, indicating that their transient overexpression could not trigger anthocyanin accumulation in the blueberry fruit pericarps.

## 4. Discussion

According to their R repeat numbers and sequences, MYBs could be classified into four major types, i.e., 1R-MYB/MYB-related, R2R3-MYB, 3R-MYB and 4R-MYB [25]. In plants, R2R3-MYBs and 1R-MYBs are the major types of MYBs. It was frequently discovered that most of the ABRMs belong to the R2R3 type, followed by the 1R type [62]. In this study, we identified fifteen ABRMs from the blueberry genome data through the homologous protein sequence alignment method. Among these ABRMs, twelve were R2R3-MYBs and three were 1R-MYBs. The subcellular localization prediction results showed that almost all of these ABRMs were nucleus-localized, which was consistent with the localization characteristics of the transcription factors. The gene structure analysis revealed that the exon number of blueberry *ABRMs* varied greatly, ranging between one and eight. The gene expression analysis showed that only *VcMYB6*, *VcMYB23*, *VcMYB*, *VcMYBL2* and *VcPH4* were expressed highly in fruits at different ripening stages, suggesting that they may play more important roles in regulating anthocyanin biosynthesis in blueberry.

MYBs function in regulating anthocyanin biosynthesis by associating with bHLH, WD40 and anthocyanin biosynthesis structural genes. Wang et al. [49] reported that exogenous cytokinin treatment could induce anthocyanin accumulation in red-fleshed apple callus by promoting the expression of *MdDFR*, *MdUFGT*, *MdMYB10* and *MdbHLH3* genes, and by suppressing the expression of *MYBL2*. Moreover, they found that MdMYBL2 could interact with MdbHLH3. In the callus overexpressing *MdMYBL2*, anthocyanin accumulation decreased, and the expression levels of *MdDFR*, *MdUFGT*, *MdMYB10* and *MdbHLH3* were all significantly inhibited. *Actinidia arguta* AaMYBC1 could interact with AabHLH42. The virus-induced gene silencing of *AaMYBC1* resulted in decreased anthocyanin accumulation and the reduced expression of anthocyanin biosynthesis structural genes in ‘HB’ kiwifruit [57]. Eggplant SmMYB86, a negative regulator of anthocyanin biosynthesis, can inhibit the expression of *SmCHS*, *SmF3H* and *SmANS* by binding to their promoters [63]. The down-regulation of *SmMYB86* resulted in anthocyanin content reduction and *SmCHS*, *SmF3H* and *SmANS* gene expression up-regulation, and its overexpression resulted in a significant decrease in the anthocyanin content in eggplant.

In this study, we predicted the protein-protein interaction network of blueberry ARBMs. According to their interacting proteins, it was predicted that the functions of these MYBs varied. VcCPC, VcMYB6 and VcMYBATV were found to be highly homologous to AtCPC, AtMYBR1 and AtTT2, respectively. AtCPC is a MYB that negatively regulates anthocyanin accumulation [64], suggesting that VcCPC might play a negative role in regulating the anthocyanin biosynthesis in blueberry. AtMYBR1 (also called AtMYB44) is involved in the abiotic stress responses of *A. thaliana* and exhibited a negative regulatory role in anthocyanin biosynthesis. Sweet potato IbMYB44 could interact with IbMYB340 and IbNAC56a/b, thereby inhibiting the formation of the MYB340-BHLH2-NAC56 complex and negatively regulating the accumulation of anthocyanins [52]. Apple MdMYB6 can bind to the promoters of *MdANS* and *MdGSTF12*, and the overexpression of *MdMYB6* in red apple callus would reduce anthocyanin accumulation and inhibit the expression of *MdANS* and *MdGSTF12* [51]. RCAR3, the interacting protein of AtMYBR1, is an ABA receptor regulator and plays a pivotal role in activating ABA signaling [65], suggesting that VcMYB6 might play a role in the ABA-regulated anthocyanin biosynthesis in blueberry. AtTT2 (AtMYB123) is mainly responsible for the biosynthesis regulation of tannins, such as proanthocyanidins [66]. VcMYB23 and VcRVE8 are homologous to AtMYB15 and AtRVE8, respectively. In Arabidopsis, the AtMYB15 interacts with AtICE1, and AtRVE interacts with AtLNK2 and AtLNK2. ICE1 plays an important role in plants’ responses to low temperatures [67], while LNK1 and LNK2 play roles in the integrated regulation of light signal responses and circadian regulation [68,69], and they can also act as corepressors of phenylpropanoid metabolism by interacting with MYB [70], suggesting that VcMYB23 and VcREV8 might be involved in the low temperature and light triggered anthocyanin biosynthesis in blueberry, respectively.

It is worth noting that VcMYB shared the highest similarity with one reported blueberry MYB (VcMYBA) [32] and was identified as a homologous protein of many positive anthocyanin regulatory MYB proteins, such as Arabidopsis AtMYB114, AtMYB90 (PAP2), AtMYB75 (PAP1) and AtMYB113, *Helianthus tuberosus* HtMYB2, monkeyflower PELAN, *Eutrema salsugineum* EsMYB90, *Freesia hybrida* FhPAP1, grape hyacinth MaAN2 and apple MdMYB10. In Arabidopsis, the AtMYB75/90/113/114 members of the SG6 subfamily of the MYB family have been proven to be involved in the regulation of anthocyanin biosynthesis; the SG7 subfamily members AtMYB11/12/111 are involved in the flavonol biosynthesis regulation; the AtMYB5/123 are involved in the regulation of tannin biosynthesis, and AtMYB3/4/7/32 encode the transcriptional repressors [36]. The expression levels of *HtMYB2* in the root, stem, leaf and tuber epidermis of the red-skinned tubers variety ‘QY1’ are higher than those of the white-skinned tubers variety ‘QY3’. The heterologous overexpression of *HtMYB2* in tobacco activates the anthocyanin biosynthesis pathway and accumulated pigments in leaves [37]. PyMYB114 and PyMYB10 can activate anthocyanin biosynthesis by interacting with PybHLH3; PyWRKY26 and PybHLH3 can synergistically target the promoter of *PyMYB114* and participate in the regulation of anthocyanin biosynthesis and transportation [71]. The co-expression of the *PalbHLH1* and *PalMYB90* genes in *Populus alba* could improve its disease resistance by promoting flavonoid synthesis [72]. The overexpression of *MdMYB90-like* upregulated the expression of the anthocyanin biosynthesis related structural genes and regulatory genes (including *MdCHS*, *MdCHI*, *MdANS*, *MdUFGT*, *MdbHLH3* and *MdMYB1*) and induced the accumulation of pigments in transgenic callus and pericarps [73]. The overexpression of onion *MYB1* can restore anthocyanin accumulation and the cyanic petal phenotype of the *A. majus R2R3-MYB* mutant, and heterologous co-expression of *MYB1* and *bHLH* can promote ectopic red pigmentation in garlic plants [74]. VcMYB is highly homologous to AtMYB114, which is a SG6 subfamily member of the Arabidopsis MYB family that positively regulates anthocyanin biosynthesis. Moreover, based on our transcriptome data, we found that the expression levels of *VcMYB* in RF, PF and BF were the highest among all members. Our qRT-PCR results also revealed that *VcMYB* was expressed significantly higher in the fruits at the red, purple and blue stages, accounting for 1.79-fold, 2.1-fold and 1.75-fold of that in GF, respectively. These results suggested that *VcMYB* may be a pivotal positive regulator of anthocyanin biosynthesis in blueberry. To verify the function of *VcMYB* in anthocyanin biosynthesis in blueberry, it was further cloned and functionally analyzed. Similar to sweet potato *IbMYB1* [75], the *VcMYB* also has three transcripts. The transient transformation results showed that only the longest *VcMYB* transcript, *VcMYB-1*, could promote anthocyanin accumulation in young blueberry fruit pericarps.

These results have demonstrated that the expression of anthocyanin regulatory *MYBs* was greatly influenced by light, ABA and MeJA [76,77,78,79]. Consistently, in this study, we identified many light-, ABA- and MeJA-elements in the promoters of blueberry *ABRMs*. Light quality and quantity greatly and widely affect the biosynthesis of anthocyanins or flavonoids in various plants [79,80]. UV-B treatment could upregulate the expression of the *HY5* gene that encodes the UV receptor at the green fruit stage, and HY5 promoted anthocyanin accumulation by upregulating the expression of *VcMYBA1*, while inhibiting the expression of *VcMYBC2* [30]. However, the expression of *VcMYBC2* was induced by UV-B treatment in the mature fruits, which could inhibit the excessive accumulation of anthocyanins. JA is an important signal for the biosynthesis of plants’ secondary metabolites, and its induction role in anthocyanin accumulation has also been found in many plants [77]. Exogenous JA treatment can activate the expression of anthocyanin biosynthesis related structural genes and regulatory transcription factor genes, and enhance anthocyanin accumulation in Arabidopsis under far-red light [81]. In apple, JA can induce anthocyanin and proanthocyanin accumulations by regulating the JAZ-TRB1-MyB9 complex [78]. In addition, MeJA treatment could induce the expression of *MdMYB9* and *MdMYB11*, whose overexpression in apple callus could improve anthocyanin and proanthocyanin accumulation, and this promotion effect could be further enhanced by MeJA [82]. ABA is considered to be one of the most important positive regulators of the ripening of non-climacteric fruits and anthocyanin biosynthesis [32,83,84]. Karppinen et al. [85] found that exogenous ABA treatment could promote the accumulation of anthocyanins in *V. myrtillus*. Han et al. [32] found that the expression levels of *CHS*, *CHI*, *DRF*, *LDOX*/*ANS* and some other anthocyanin biosynthesis related genes were significantly up-regulated in blueberries when treated with ABA at the late ripening stages, and were highly positively correlated with the anthocyanin content. Consistently, in our present study, we found that 16 of the 19 identified blueberry *ABRM* genes contained the ABA-responsive ABRE element in their promoters, indicating that ABA have a significant function in influencing the expression of blueberry *ABRMs* and in regulating anthocyanin biosynthesis.

## 5. Conclusions

In this study, we identified fifteen candidate ABRM proteins from blueberry. Among them, twelve were R2R3-MYBs and three were 1R-MYBs, which could be well supported by their conserved motif types and numbers. With the exception of VcRVE8, which was localized in the chloroplast and nucleus, all of the blueberry ABRMs were predicted to be nucleus-localized. The exon numbers of the blueberry *ABRM* genes varied significantly. The promoters of *ABRMs* contain a large number of light-, ABA- and MeJA-responsive elements, indicating that the influences of these factors on anthocyanin accumulation were achieved, at least partially, by regulating the expression of the *ABRM* genes. *VcMYB* was highly expressed in blueberry fruits at the late ripening stages. This gene had three transcripts; however, only the transient overexpression of its longest transcripts (*VcMYB-1*) could promote anthocyanin accumulation in young blueberry fruits. Our study will provide a basis for the applications of the blueberry anthocyanin biosynthesis related *MYB* genes, and will lay the foundation for the high-anthocyanin aimed blueberry selection and breeding in the future.

## Figures and Tables

**Table 1:** Information for the primers used in this study.

Target Gene	Primer Name	Primer Sequence	Target Length (bp)	Annealing Temperature (°C)	Applications
*VcMYB*	VcMYB-F	ATGGACATAGTTCCATTGGGAGTGA	798	59	Gene cloning
	VcMYB-R	TAAAATATCCCAAAGGTCCACATTGTC			
	VcMYB-InF	ACGGGGGACTCTAGAGGATCCATGGACATAGTTCCATTGGGAGTGA	786/704/568	60	Vector construction
	VcMYB-InR	GCTCACCATCGCTGCACTAGTTAAAATATCCCAAAGGTCCACATTGTC			
	VcMYB-qF	TCCATTGGGAGTGAGAAAGG	115	60	qRT-PCR
	VcMYB-qR	CAATCCTGCCCTGTAAGGAA			
*VcMYB6*	VcMYB6-qF	CTCTCCTCAGGTGGAGCATC	164	60	qRT-PCR
	VcMYB6-qR	TTCCTCTTGAGCGTGGAGTT			
*VcMYB2*	VcMYBL2-qF	TCAAAATCCACGTCCCTCTC	92	60	qRT-PCR
	VcMYBL2-qR	CATTCTCCGCTAGCTTGGTC			
*VcMYB23*	VcMYB23-qF	TGTTGGGAAACAGATGGTCA	89	60	qRT-PCR
	VcMYB23-qR	TTTCAAGTGGGTGTGCCATA			
*GAPDH*	GAPDH-qF	ACTACCATCCACTCTATCACCG	116	59	qRT-PCR
	GAPHD-qR	AACACCTTACCAACAGCCTTG			

**Table 2:** The identified blueberry ABRM proteins.

Protein Name	Gene ID	Homologous Protein Name	Homologous Gene ID	Similarity (%)	References
VcMYB	VaccDscaff1486-snap-gene-0.3	VcMYBA	MH105054	93.40	[32]
		AtMYB114	At1G66380	74.36	[36]
		HtMYB2	MN887536	62.25	[37]
		PELAN	KJ011144	59.87	[38]
		EsMYB90	XP_006391393	59.35	[39]
		AtMYB90(PAP2)	At1G66390	58.97	[36]
		AtMYB75(PAP1)	At1G56650	58.71	[36]
		FhPAP1	MT210093	56.86	[40]
		MaAN2	KY781168	56.86	[41]
		AtMYB113	At1G66370	56.77	[36]
		MdMYB10	EU51829.2	54.36	[42]
VcMYB12	VaccDscaff43-snap-gene-6.43	AtMYB12	At2G47460	66.86	[46]
		AtMYB11	At3G62610	52.17	[46]
		AtMYB111	At5G49330	50.19	[46]
VcMYB123	VaccDscaff34-augustus-gene-10.31	AcMYB123	MH643776	72.33	[47]
		MdMYB9	MDP0000210851	71.43	[48]
VcMYBL2	VaccDscaff28-augustus-gene-197.19	MdMYBL2	NP_001281006.1	65.29	[49]
		SmelMYBL1	MN855525	51.61	[50]
VcMYB6	VaccDscaff31-processed-gene-75.6	MdMYB6	DQ074461	55.26	[51]
		IbMYB44	itf03g30290.t1	51.46	[52]
VcPL	VaccDscaff13-augustus-gene-110.26	OsPL	LOC_Os05g48010.1	75.19	[53]
VcMYB24L	VaccDscaff30-augustus-gene-338.25	MdMYB24L	XM_008343218.2	75.00	[54]
VcRVE8	VaccDscaff34-augustus-gene-308.23	PbRVE8	XP_009342285.1	68.22	[55]
VcCPC	VaccDscaff41-snap-gene-184.30	AtCPC	At2G46410	67.06	[56]
VcMYBC1	VaccDscaff32-augustus-gene-55.27	AaMYBC1	MN249175	57.41	[57]
VcMYB23	VaccDscaff46-processed-gene-168.9	MdMYB23	MDP0000230141	55.15	[58]
VcMYB340	VaccDscaff16-snap-gene-84.41	IbMYB340	itf12g05820.t1	55.02	[52]
VcPH4	VaccDscaff39-augustus-gene-189.28	CsPH4	Cs9g03070	54.14	[59]
VcMYBATV	VaccDscaff1069-augustus-gene-0.8	SlMYBATV	Solyc07g052490.4.1	53.57	[60]
VcMYB85	VaccDscaff36-augustus-gene-8.19	SiMYB85	Seita.4G086300	50.72	[61]

**Table 3:** Basic physicochemical properties of the identified blueberry ABRMs. CDS: coding sequence; PI: isoelectric point; MW: molecular weight; PI: isoelectric point.

Gene Name (ID)	CDS Length/bp	Protein Size/aa	MW/Da	PI	Instability Index	GRAVY	Subcellular Localization
*VcMYB* (VaccDscaff1486-snap-gene-0.3)	822	273	31,294.22	6.01	41.78	−0.749	Nucleus
*VcMYB12* (VaccDscaff43-snap-gene-6.43)	1083	360	39,534.64	6.68	54.28	−0.603	Nucleus
*VcMYB123* (VaccDscaff34-augustus-gene-10.31)	828	275	31,260.2	7.55	52.05	−0.705	Nucleus
*VcMYBL2* (VaccDscaff28-augustus-gene-197.19)	702	233	26,419.91	8.40	51.94	−0.779	Nucleus
*VcMYB6* (VaccDscaff31-processed-gene-75.6)	1080	359	39,018.97	6.26	61.47	−0.498	Nucleus
*VcPL* (VaccDscaff13-augustus-gene-110.26)	903	300	33,807.74	5.90	55.58	−0.702	Nucleus
*VcMYB24L* (VaccDscaff30-augustus-gene-338.25)	573	190	21,953.61	6.16	54.33	−0.854	Nucleus
*VcRVE8* (VaccDscaff34-augustus-gene-308.23)	942	313	33,898.29	7.78	46.57	−0.462	Chloroplast; Nucleus
*VcCPC* (VaccDscaff41-snap-gene-184.30)	297	98	11,752.33	9.72	79.67	−1.014	Nucleus
*VcMYBC1* (VaccDscaff32-augustus-gene-55.27)	798	265	30,057.3	8.57	58.02	−0.68	Nucleus
*VcMYB23* (VaccDscaff46-processed-gene-168.9)	738	245	27,819.19	6.41	36.10	−0.668	Nucleus
*VcMYB340* (VaccDscaff16-snap-gene-84.41)	744	247	28,372.85	7.66	54.91	−0.855	Nucleus
*VcPH4* (VaccDscaff39-augustus-gene-189.28)	1086	361	40,095.85	8.72	48.94	−0.719	Nucleus
*VcMYBATV* (VaccDscaff1069-augustus-gene-0.8)	222	73	8407.08	4.31	61.99	−0.922	Nucleus
*VcMYB85* (VaccDscaff36-augustus-gene-8.19)	912	303	33,738.76	8.37	60.02	−0.775	Nucleus

## Data Availability

All data are available in this article.

## Supplementary Materials

The following supporting information can be downloaded at: https://www.mdpi.com/article/10.3390/cimb45010027/s1. Figure S1: Phylogenetic analysis result of anthocyanin biosynthesis related MYB proteins from blueberry and some other plant species.
