# Supplementary figures and images for: Characterization of Highbush Blueberry (Vaccinium corymbosum L.) Anthocyanin Biosynthesis Related MYBs and Functional Analysis of VcMYB Gene
Source: Curr Issues Mol Biol. 2023 Jan 3;45(1):379–99. doi: 10.3390/cimb45010027 (PMC9857026; doi:10.3390/cimb45010027)

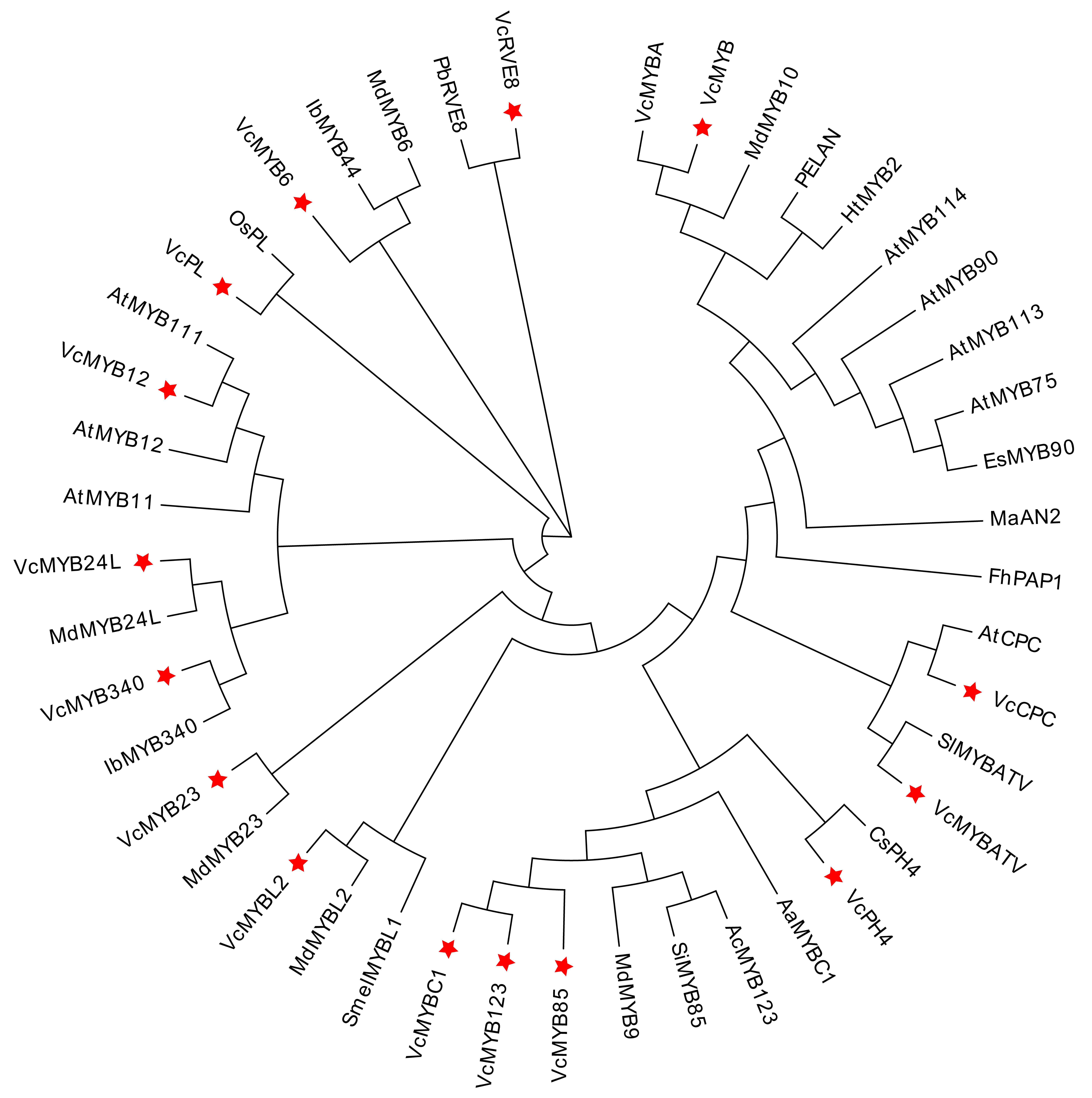

Supplement: Supplementary file 1 [file cimb-45-00027-s001.zip › Figure S1.tif]
